# Disruption of cardiovascular circadian rhythms in mice post myocardial infarction: relationship with central angiotensin II receptor expression

**DOI:** 10.14814/phy2.12210

**Published:** 2014-11-20

**Authors:** Tarek M. Mousa, Alicia M. Schiller, Irving H. Zucker

**Affiliations:** 1 Department of Cellular and Integrative Physiology, University of Nebraska Medical Center, Omaha, Nebraska

**Keywords:** Cardiac dysfunction, cardiovascular reflex, diurnal variability, oxidative stress

## Abstract

Angiotensin II (Ang II) is well known to participate in the abnormal autonomic cardiovascular control that occurs during the development of chronic heart failure (CHF). Disrupted cardiovascular circadian rhythm in CHF is also well accepted; however, the mechanisms underlying and the role of central Ang II type 1 receptors (AT1R) and oxidative stress in mediating such changes are not clear. In a post myocardial infarction (MI) CHF mouse model we investigated the circadian rhythm for mean arterial pressure (MAP), heart rate (HR), and baroreflex sensitivity (BRS) following MI. The cardiovascular parameters represent the middle 6‐h averages during daytime (6:00–18:00) and nighttime (18:00–6:00). HR increased with the severity of CHF reaching its maximum by 12 weeks post‐MI; loss of circadian HR and BRS rhythms were observed as early as 4 weeks post‐MI in conjunction with a significant blunting of the BRS and an upregulation in the AT1R and gp91^phox^ proteins in the brainstem. Loss of MAP circadian rhythm was observed 8 weeks post‐MI. Circadian AT1R expression was demonstrated in sham animals but was lost 8 weeks following MI. Losartan reduced AT1R expression in daytime (1.18 ± 0.1 vs. 0.85 ± 0.1; *P* < 0.05) with a trend toward a reduction in the AT1R mRNA expression in the nighttime (1.2 ± 0.1 vs. 1.0 ± 0.1; *P* > 0.05) but failed to restore circadian variability. The disruption of circadian rhythm for HR, MAP and BRS along with the upregulation of AT1 and gp91^phox^ suggests a possible role for central oxidative stress as a mediator of circadian cardiovascular parameters in the post‐MI state.

## Abstract (precis)

Increases in central angiotenisn II signaling provide a driving force for sympatho‐excitation in heart failure. In this study, we show a loss of circadian variability in angiotensin type 1 receptor expression in the brainstem of mice post myocardial infarction. These changes correlate with a loss of cardiovascular circadian variability. These data suggest that sympatho‐ excitation may be increased in the post‐MI state at times when sympathetic outflow is normally reduced.

## Introduction

It is now widely accepted that various cardiovascular parameters change with a 24‐h cycle. For example, blood pressure (BP), heart rate (HR), cardiac output, and stroke volume are higher in the active phase than in the rest phase (Millar‐Craig et al. 1978; Lemmer 2006). It is also well established that rhythms for BP and HR are controlled by an endogenous circadian oscillating system (Takezawa et al. 1994). Following acute myocardial infarction (MI) patients exhibit abnormal sympatho‐vagal balance (Milicevic 2005; Khandoker et al. 2010) and impairment in arterial baroreflex function (Farrell et al. 1992; Grassi et al. 1999; De Ferrari et al. 2007). These autonomic changes may act as compensatory mechanisms that maintain cardiac output following MI; however, these same compensatory modulations may also contribute to worsening of cardiac function over time (Dzau 1993; Svanegaard et al. 1993). In humans, sympatho‐vagal imbalance following an MI exhibits a diurnal rhythm being the greatest during morning hours which predisposes to potentially fatal cardiac arrhythmias and sudden cardiac death, (Millar‐Craig et al. 1978; Sami 1991; Kinoshita et al. 2005; Taylor et al. 2011; Jeyaraj et al. 2012) especially in patients with preexisting cardiovascular disease. In addition, arterial baroreflex sensitivity (BRS) also exhibits circadian variability (Millar‐Craig et al. 1978; Taylor et al. 2011). Decreases in baroreflex sensitivity that may occur when sympathetic nerve activity rises may contribute to arrhythmogenesis in chronic heart failure (CHF).

Angiotensin II (Ang II) modulates sympathetic outflow and blunts the baroreflex by acting centrally at discrete sites such as the nucleus tractus solitarius (NTS) (Wang et al. 2004, 2006; Tan et al. 2007), the paraventricular nucleus (PVN) (Li and Ferguson 1993; Han et al. 2005), and the rostral ventrolateral medulla (RVLM) (Head 1996; Matsuura et al. 2005; Zucker et al. 2009) primarily by acting through the angiotensin II Type 1 receptor (AT1R). In cardiovascular disease states such as CHF, it is now clear that the activation of NADPH oxidase‐derived reactive oxygen species are increased in the brain and that increase is driven, in part, by Ang II (Gao et al. 2004; Zimmerman et al. 2004). Increases in the central mRNA and protein expression of AT1R and NADPH oxidase subunits (p40^phox^, p47^phox^, and gp91^phox^) have been demonstrated in animals with MI and CHF (Gao et al. 2004). While several studies have shown an upregulation in these proteins, it is still not clear if these changes occur early or late in the development of CHF. In addition, changes in AT1R protein expression during a 24‐h cycle may contribute to sympatho‐excitation during the active period. The contribution of these circadian variations to baroreflex function and sympatho‐excitation in CHF is not known. Our laboratory has previously shown a positive feedback mechanism in the control of the AT1R, where Ang II through the AT1R evokes a transcriptional upregulation of the AT1R, a clearly pathological event that may contribute to sympatho‐excitation and worsening of the CHF state (Liu et al. 2008; Mitra et al. 2010; Haack et al. 2012).

Given the above, this study was undertaken to (1) track hemodynamic changes (over 24 h) post‐MI in a mouse model; and (2) to correlate such changes with the expression of AT1R and NADPH oxidase in the central nervous system. We hypothesized that central AT1R expression would exhibit a blunted circadian rhythm in the post‐MI state. We also hypothesized that blockade of AT1R in the post‐MI state would restore cardiovascular circadian rhythms and reduce sympatho‐excitation.

## Methods

All procedures have been approved by the Institutional Animal Care and Use Committee of the University of Nebraska Medical Center and conformed with the NIH Guide for the Care and Use of Experimental Animals. Male C57BL/6 mice (*n* = 99) all weighing approximately 30 g were studied post‐MI or after a sham operation at various time intervals: 2, 4, 8, 12, and 16 weeks.

### Myocardial infarction procedure

Myocardial infarctions were produced as described previously Xiao et al. (2011). In brief, mice were anesthetized by intramuscular injection with a mixture of ketamine (9 mg/100 g), Acepromazine (4 mg/100 g), atropine (0.06 mg/100 g), and prepared for sterile surgery. Animals were intubated with a 20G catheter and ventilated with a mixture of O_2_ and room air, using a model 845 ventilator (Harvard Apparatus, Holliston, MA). The stroke volume was set at 0.3 mL and the respiratory rate was 125 breaths/min. After a left anterior thoracotomy, the heart was exposed and the location of the left coronary artery (LCA) on the surface of LV anterior wall was identified. A 7–0 silk suture (Ethicon) was placed around the LCA and was tightened. Occlusion of the LCA was confirmed by change in the color at the involved LV wall. Lungs were expanded to displace air before the chest was closed; the mouse was then extubated. Mice were allowed to recover from the surgery in an oxygen‐filled chamber over a heating pad. Sham‐operated mice underwent similar surgery without occlusion of the LCA.

### Hemodynamic recordings

Radiotelemetry pressure transducers (TA11PA‐C10 Data Sciences International, St. Paul, MN) were implanted as described previously Xiao et al. (2011). The catheter of the pressure telemetry was implanted in a retrograde fashion in the left carotid artery. The transmitter unit was implanted under the skin on the back of the animal at the time of closure. After 1 week of recovery, hemodynamic recordings were acquired over 24 h in the conscious, unrestrained state. Daytime (lights on 06:00–18:00) and nighttime (lights off 18:00–06:00) values were obtained by averaging 5 min of continuous recording every hour using the “macro” extension of Chart 5.0 software (ADInstruments, Inc.; Colorado Springs, Co.).

### Baroreflex sensitivity assessment

The spontaneous baroreflex technique was used to assess the baroreflex sensitivity (BRS) as described previouslyParlow et al. (1995). In brief, arterial pressure pulses acquired from telemetry were analyzed by Hemolab software kindly provided by Dr. Harold Stauss (University of Iowa) (Hemolab 0.9.0). A minimum R value of 0.8 was used for data (pulse interval vs. arterial systolic pressure) inclusion. The program detected three or more adjacent sequences where the blood pressure signals (i.e., pulses) were either up‐sloping or down‐sloping that was concurrently accompanied by a decrease or an increase in the subsequent pulse rate (i.e., HR), respectively. Regression slopes plotting HR against BP were determined and the average slope was termed the BRS for that data collection period.

### Echocardiographic measurements

Cardiac function was assessed while mice were under light (0.2–0.5%) isoflurane anesthesia by echocardiography (VEVO 770; Visual Sonics Inc., Toronto, Canada) at different time intervals post‐MI or sham operation (at 2, 4, 8, 12, and 16 weeks). Left ventricular end‐diastolic (LVED) and end‐systolic diameters (LVSD) were measured and the ejection fraction (EF) was calculated.

### Water intake and urine output

After implantation of the radiotelemetry units at different time periods post‐MI or sham, mice were weighed and placed individually in metabolic cages (NALGENE Labware, Rochester, NY). After an acclimation period of 48 h, basal 24‐h urine collection with free access to a standard pulverized mouse diet and water was taken. The water intake was measured and urine output was collected under mineral oil.

### Western blot analysis of AT1R and gp91^phox^

Upon termination of the metabolic and hemodynamic recordings, mice were euthanized and the brains rapidly removed and frozen on dry ice. The entire brainstem (pons to spinal cord) and hypothalamus (optic chiasm to most anterior portion of the pons) was used for biochemical analysis. The tissue was homogenized with a homogenizer in radioimmunoprecipitation assay buffer. Protein extraction from homogenates was used for western blot analysis for the mouse AT1R and the NADPH oxidase subunit, gp91^phox^. The protein concentration was measured using a protein assay kit (Pierce; Rockford, IL). Samples were adjusted to the same concentration of protein, mixed with equal volumes of 2× 4% SDS sample buffer, and then boiled for 5 min following by loading on the 7.5% SDS‐PAGE gel (5 *μ*g protein/30 *μ*L per well) for electrophoresis using Bio‐Rad (Hercules, CA) mini gel apparatus at 40 mA/each gel for 45 min. The fractionized proteins on the gel were electrophoretically transferred onto the PVDF membrane (Millipore, Billerica, MA) at 300 mA for 90 min. The membrane was probed with primary antibody (mouse anti‐human AT1R polyclonal antibody; Santa Cruz Biotechnology, Dallas, TX). The gp91^phox^ antibody was also purchased from Santa Cruz.

### qRT‐PCR analysis of AT1R and gp91^phox^

Total RNA was extracted from the entire brainstem and hypothalamus with a RNeasy Mini KitTotal RNA Isolation System (Qiagen, Valencia, CA), after which cDNA was synthesized by means of Maloney murine leukemia virus reverse transcriptase (Invitrogen Life Technologies, Carlsbad, CA). PCR amplification was performed by means of a PTC‐100 Programmable Thermal Controller (MJ Research, Waltham, MA) as follows: 1 cycle at 95°C for 15 min, followed by 35 cycles of 94°C for 45 sec, 55°C for 45 sec, and 72°C for 1 min. The bands were analyzed using UVP BioImaging Systems (Upland, CA). The primer sequences for AT1R and gp91^phox^ mRNA were as follows:

AT1R
  - Primer (S): GTGTTCCTGCTCACGTGTCT
  - Primer (AS): GATGATGCAGGTGACTTTGG
  - Probe: ATGAAGTCTCGCCTCCGCCG
gp91
  - Primer (S): GTGAGAGGTTGGTTCGGTTT
  - Primer (AS): CACCTCCATCTTGAATCCCT
  - Probe: CACCAAGGTGGTCACCCACCC
GAPDH
  - Primer (S): ACAACTTTGGCATTGTGGAA
  - Primer (AS): GATGCAGGGATGATGTTCTG
  - Probe: CATGCCATCACTGCCACCCA

### Cardiac and lung tissue analysis

Upon euthanasia, the heart was removed, the left ventricle dissected, and the infarct area measured from a digital image. Infarct size was represented as a percent of the left ventricle. Heart weight, lung wet, and dry weights were determined to estimate lung water content (see below).

### Losartan infusion

Losartan was infused in mice 8 weeks post‐MI using an osmotic minipump (Alzet Durect, Cupertino, CA, model 2001). The minipump was implanted through a small incision under the skin on the back using local anesthesia. Losartan was infused for 5 days at a dose of 1 *μ*g/g.

### Experimental protocol

Mortality rate following the coronary ligation procedure approached 40–50%. In addition, two of the sham‐operated animals died following the procedure. Following either sham surgery or coronary ligation and at various time intervals (2, 4, 8, 12, and 16 weeks) telemetry units were implanted 5 days before acquiring any hemodynamic recordings. Blood pressure recordings were acquired by recording for 5 min every hour over 24 h for two consecutive days. At the end of the experiment, animals were euthanized with pentobarbital sodium (150 mg/kg, IP). Animals were euthanized at 12:00 h (daytime) and 0:00 h (nighttime). Brains were taken and flash frozen on dry ice. The heart was harvested and weighed. The LV infarct area was determined digitally using SigmaScan software (Sysstat Software, Inc. San Jose, CA) where the infarct area was represented as a percent of the LV. Lungs were weighed wet then dried overnight in an 80°C oven, the dry weights were determined and the water content was calculated.

Acquired data were then analyzed by assessing the spontaneous BRS by uploading the data to analysis software (Hemolab 0.9.0) where sequences were detected and the mean regression curve was taken as the BRS for that time period. During the 6 h in midday (9:00–15:00) and midnight (21:00–03:00), we acquired six 5‐min intervals that were analyzed and the results averaged for the daytime and the nighttime periods. MAP and HR were also averaged during the same time periods.

In the second set of experiments, we assessed daytime/nighttime AT1R expression. Mice were euthanized and the brains harvested and fresh frozen for subsequent analysis. For daytime expression, animals were euthanized at 12:00. For nighttime expression, animals were euthanized at 00:00. In a subgroup of animals, the AT1R antagonist losartan was infused using osmotic minipumps. Telemetry units in this set of experiments were implanted 5 days before implanting the osmotic minipumps. Animals receiving losartan were also euthanized at similar day and nighttime hours.

### Statistical analysis

Data are shown as means ± SEM; statistical significance was evaluated by a paired Student's *t*‐test and one‐way ANOVA using Bonferroni post hoc test where appropriate. Results were considered significant when *P* < 0.05.

## Results

### Cardiovascular parameters and metabolism

Table 1 summarizes cardiac function, water intake, urine output, and relative heart weight in sham and MI mice. In addition, lungs were weighed wet and dry, as a marker of pulmonary congestion, at different time intervals in the sham and MI animals. Ejection fraction was significantly decreased at 4 weeks post‐MI compared to sham mice. Ejection fraction remained significantly reduced in MI mice for the remainder of the observation period. Mice began to retain fluid as denoted by a reduction in renal output along with an increase in water intake that did not achieve statistical significance until 12 weeks post‐MI. Heart weight (HW)/body weight (BW) ratio was significantly higher in MI mice, reflecting hypertrophy of the surviving myocardium. The HW/BW ratio was significantly increased (compared to sham) at 4 weeks post‐MI. This coincided with a significant decrease in EF. Heavier lungs, denoting pulmonary congestion, was a prominent finding in mice beginning at the 8‐week time point, post‐MI. Sham‐operated animals did not exhibit any change in these parameters over the observation period.

**Table 1.** Baseline cardiac function and fluid balance in sham and MI mice over time.

	2‐Week Sham (*n* = 4)	2‐WeekMI (*n* = 7)	4‐Week Sham (*n* = 5)	4‐Week MI (*n* = 8)	8‐Week Sham (*n* = 9)	8‐Week MI (*n* = 9)	12‐Week Sham (*n* = 5)	12‐Week MI (*n* = 11)	16‐Week Sham (*n* = 4)	16‐Week MI (*n* = 6)
EF (%)	72 ± 1	67 ± 2	78 ± 1	54 ± 3*	75 ± 2	54 ± 5*	77 ± 1	57 ± 4*	75 ± 1	59 ± 1
MI (%LV)		38 ± 1		40 ± 1		39 ± 1		40 ± 1		40 ± 1
Water intake (mL/day)	5.6 ± 0.2	5.5 ± 0.1	5.4 ± 0.2	6.9 ± 0.4	5.7 ± 0.2	6.8 ± 0.3	5.5 ± 0.7	7.3 ± 0.3*	5.7 ± 0.2	7.7 ± 0.2*
Urine output (mL/day)	1.7 ± 0.1	1.5 ± 0.1	1.7 ± 0.1	1.4 ± 0.1	1.7 ± 0.1	1.1 ± 0.1	1.6 ± 0.1	1.1 ± 0.1*	1.6 ± 0.2	1.0 ± 0.1*
HW/BW (mg/g)	9 ± 1	8 ± 0.3	8 ± 1	13 ± 1*	7 ± 1	11 ± 1*	7 ± 1	10 ± 1*	8 ± 1	11 ± 0.2*
Lung Wet Wt (g)	0.2 ± 0.01	0.2 ± 0.01	0.3 ± 0.01	0.3 ± 0.02	0.21 ± 0.01	0.32 ± 0.02*	0.24 ± 0.03	0.32 ± 0.01*	0.24 ± 0.03	0.33 ± 0.01*
Body Weight (g)	24.5 ± 1.0	23.6 ± 0.7	27.3 ± 1.0	24.8 ± 1.5	27.2 ± 0.6	27.3 ± 0.5	30.0 ± 0.6	30.2 ± 0.6	28.2 ± 1.6	28.2 ± 1.0
Lung Dry Wt (g)	0.05 ± 0.0	0.05 ± 0.0	0.05 ± 0.0	0.05 ± 0.0	0.05 ± 0.0	0.05 ± 0.0	0.05 ± 0.0	0.06 ± 0.0	0.06 ± 0.01	0.06 ± 0.01

* *P* < 0.05 compared to the corresponding sham group.

**Figure 1.**
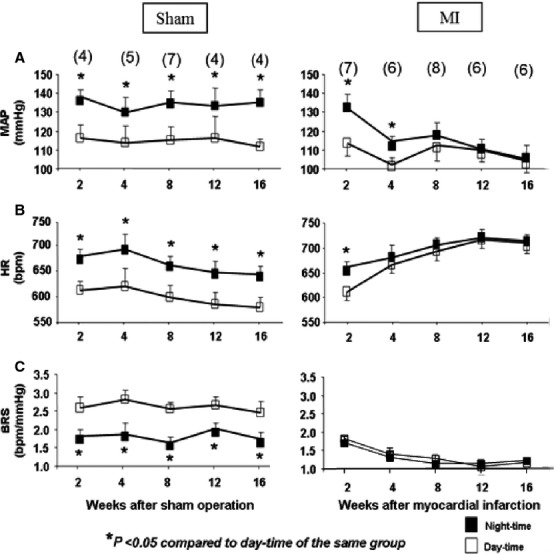
Hemodynamic and spontaneous baroreflex sensitivity for both sham and MI mice over time, taken during the daytime (open squares) and nighttime (closed squares). MAP = mean arterial pressure; HR = heart rate; BRS = baroreflex sensitivity. Numbers in parentheses are the n per group.

Figure 1 shows the time course for changes in MAP; panel A, HR; panel B, and BRS; panel C in sham animals (left panels) and in animals post‐MI (right panels) during both nighttime and daytime periods. The sham data were similar at each time period, suggesting that the circadian variability was stable over time and that any difference in MI animals was not the result of time‐dependent changes, but the result of changes taking place after MI. Sham animals exhibited day–night variability for MAP, HR, and BRS. Post‐MI mice exhibited differences between the day–night values for MAP at 2 and 4 weeks and HR at 2 weeks. This difference was lost by 8 weeks post‐MI. Therefore, from 8 to 12 weeks post‐MI, there were no dips in MAP during the daytime. This was associated with a significant drop in EF and increases in the lung weights. In MI mice, the HR increased over time suggesting sympatho‐excitation as cardiac function deteriorates. Interestingly, the day–night difference in HR was still preserved 2 weeks post‐MI. This was lost by 4 weeks (panel B). Panel C illustrates mean data for BRS which was depressed in mice as early as 2 weeks post‐MI even though cardiac function was well preserved at this time point. Moreover, the day–night variability was also lost at 2 weeks post‐MI. There was a further and significant fall in BRS at 4 weeks and for the remainder of the 16 weeks. Note the differences in the scale between sham and MI mice in panel C.

**Figure 2.**
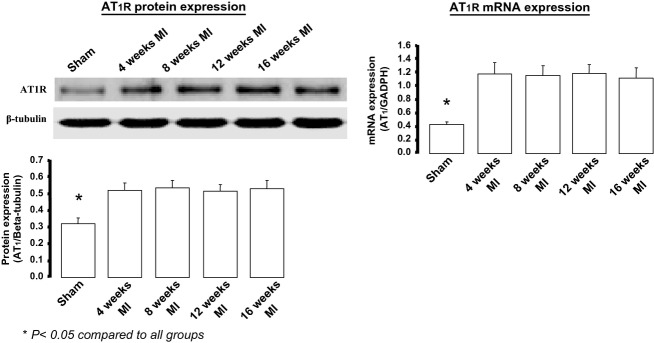
Angiotensin type 1 (AT1) receptor protein and mRNA expression in the brainstem of mice at various time periods post‐MI compared to a group of sham‐operated mice. Samples acquired during the daytime period. For sham protein and mRNA,* n* = 9. For post‐MI time points *n* = 5 per time period.

**Figure 3.**
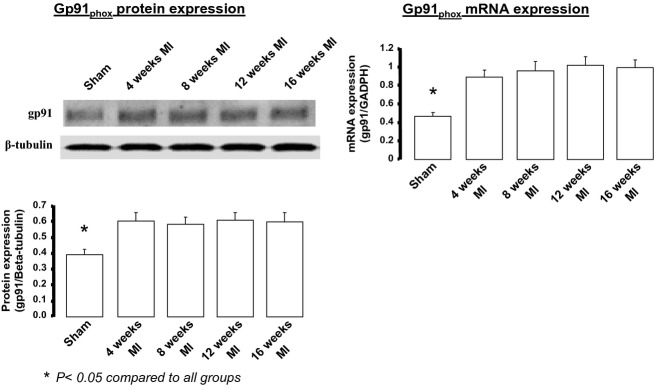
gp91^phox^ protein and mRNA expression in the brainstem of mice at various time periods post‐MI compared to a group of sham‐operated mice. Samples acquired during the daytime period. For sham protein *n* = 5. For sham mRNA,* n* = 9. For post‐MI time points *n* = 5 per time period.

Because central AT1Rs have been shown to play a role in the sympatho‐excitatory process (Reid 1992; Zucker et al. 2001, 2009), we determined if a correlation of these hemodynamic changes existed with AT1R protein and/or oxidative stress in the central nervous system, specifically in the brain stem. Figure 2 shows AT1R protein and mRNA expression at different time intervals following MI or sham surgery. The left panel shows representative western blots and mean data from each time period and from sham mice. Note we do not show 2‐week molecular data. AT1R mRNA measured by qRT‐PCR (right panel) followed a similar pattern to that of the protein expression. There was approximately a twofold increase in protein and a threefold increase in mRNA. It would appear that AT1R expression was inversely related to the EF which did not fall until 4 weeks post‐MI. Tissue from the cerebral cortex did not show any changes among groups studied (data not shown). Figure 3 shows the protein and message expression for gp91^phox^, a marker of oxidative stress, as one of the components of the Ang II‐mediated NADPH oxidase subunits. There was approximately a 0.5‐fold change in gp91^phox^ protein and message.

**Figure 4.**
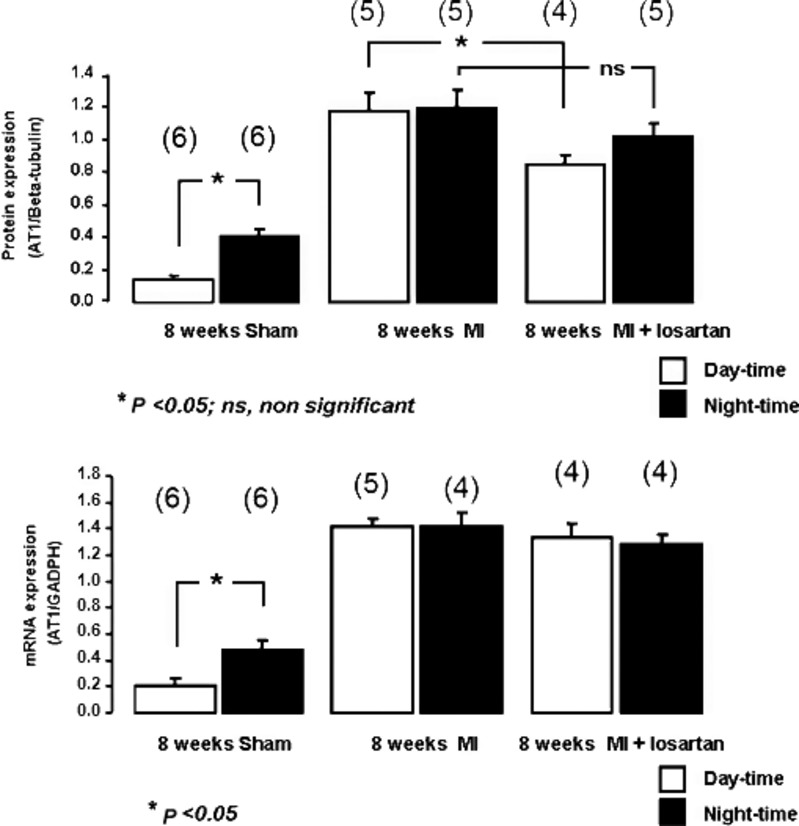
Day–night comparisons of AT1R protein and mRNA expression in sham and MI mice at 8 weeks. There was a clear increase in brainstem AT1 protein during the nighttime hours in the sham group. MI mice showed an increase in AT1 protein in both daytime and nighttime periods. Losartan treatment lowered daytime expression in MI mice, but had little effect on the nighttime expression. Numbers in parentheses are the *n* per group.

We then asked the question whether changes in central AT1Rs and gp91^phox^ could be mediating hemodynamic changes (i.e., MAP, HR, and BRS). In Fig. 4, a day–night pattern of AT1R protein expression was seen in sham animals 8 weeks following surgery. This variation was lost in the MI mice at the same time period. However, losartan infusion for 5 days failed to restore cardiac circadian rhythm, but did restore AT1R protein expression levels. Interestingly, losartan did not have an effect on levels of AT1R mRNA (Fig. 4). This suggests that normalization of the protein and also mRNA of the AT1R could be required to restore circadian rhythm.

Losartan infusion for 5 days in mice 8 weeks after MI enhanced BRS in the daytime (1.85 ± 0.1 after losartan vs. 1.26 ± 0.2 without losartan; *P* < 0.05) and nighttime (1.69 ± 0.1 after losartan vs. 1.14 ± 0.1 without losartan; *P* < 0.05), but failed to restore the circadian variability of BRS (1.85 ± 0.1 after losartan vs. 1.69 ± 0.1 without losartan; *P* > 0.05). We do not believe this was due to the improvement in cardiac function following losartan infusion as echocardiograms before and after the losartan infusion showed no differences in EF (50.8 ± 3.5% before losartan vs. 48.7 ± 5.6% after losartan; *P* > 0.05) and HW/BW ratio (12.9 ± 0.6 with losartan vs. 11.3 ± 0.6 without losartan; *P* > 0.05). Wet lung weights (0.34 ± 0.01 after losartan vs. 0.32 ± 0.02 without losartan; *P* > 0.05) were not different in the losartan‐infused mice when compared to the 8 weeks post‐MI mice without losartan. There was no significant effect on MAP in daytime (99.3 ± 7.8 mmHg after losartan vs. 111.8 ±4.6 mmHg without losartan; *P* > 0.05) or nighttime (105.7 ± 6.8 mmHg after losartan vs. 117.9 ± 4.8 mmHg without losartan; *P* > 0.05). Similarly, HR was not different in daytime after losartan (676.3 ± 13 bpm after losartan vs. 694.2 ± 12.6 bpm without losartan; *P* > 0.05) or nighttime (689.4 ± 16 bpm after losartan vs. 705.1 ±9.1 bpm without losartan; *P* > 0.05).

We measured the AT1R and gp91^phox^ protein expression in the hypothalamus, as in mice it is a technically difficult task to punch out the SCN alone. AT1R and gp91^phox^ protein expression did not exhibit day–night variability and was significantly higher in mice 8 weeks post‐MI compared to sham animals. Nevertheless, losartan failed to have significant effects on those parameters. Table 2 summarizes AT1R and gp91^phox^ protein data in shams, MI, and MI with losartan infusion 8 weeks following the sham or the coronary ligation operation in the hypothalamus.

**Table 2.** AT1R and gp91^phox^ protein in mouse hypothalamus in the sham state and 8 weeks post‐MI or post‐MI plus losartan treatment.

	8‐week sham		8‐week MI		8‐week MI + losartan	
	Daytime (*n* = 5)	Nighttime (*n* = 6)	Daytime (*n* = 5)	Nighttime (*n* = 5)	Daytime (*n* = 4)	Nighttime (*n* = 5)
AT1R protein (AT1/*β*‐tubulin)	0.3 ± 0.02*	0.4 ± 0.02*	0.8 ± 0.03	0.8 ± 0.03	0.8 ± 0.1	0.7 ± 0.02
gp91_phox_ protein (gp91/*β*‐tubulin)	0.4 ± 0.03*	0.4 ± 0.03*	0.6 ± 0.03	0.6 ± 0.03	0.6 ± 0.04	0.5 ± 0.01

* *P* < 0.05 compared to 8‐week MI and 8‐week MI + losartan.

## Discussion

The primary finding of this study is the demonstration of a correlation between the loss in cardiovascular circadian rhythms and the expression of AT1Rs in the brainstem of mice following the creation of an MI. Furthermore, a similar pattern was observed for the expression of the primary catalytic subunit of NADPH oxidase (Nox2), gp91^phox^. While no direct measurements of oxidative stress were made in this study, it is well accepted that coupling of the AT1R to Nox2 generates superoxide anion (Touyz 2004; Zucker 2006), which, in turn, can sensitize sympathetic neurons by modulation of cation channel activity (Touyz 2004; Zimmerman et al. 2005; Chan and Chan 2012). We and others (Li et al. 2006; Wang et al. 2008; Kleiber et al. 2010) have shown an important role for AT1R signaling in the generation of sympatho‐excitation in the setting of chronic heart failure. Similar observations have been made in the hypertensive state (Campese et al. 2005). While the role of central Ang II mechanisms in the sympatho‐excitatory process is well accepted, the current findings are the first, to our knowledge, to show a diurnal variability in the expression of these two proteins in the heart failure state. Given that sympathetic nerve activity and blood pressure are lower at night in humans (Narkiewicz et al. 2002) and that blood pressure dipping is lost in hypertensive and in patients with heart failure (Palatini 2004), these data are highly relevant for understanding the mechanisms related to cardiovascular disease in sympatho‐excitatory states. We hypothesized that circadian rhythms for central AT1Rs and gp91^phox^ would be lost following MI, and restored by blocking AT1R signaling since this is required for upregulation of the AT1R in the heart failure state (Liu et al. 2008).

There have been several studies implicating the AT1R in various circadian functions. Pechlivanova et al. (2010) showed a role for the AT1R in diurnal susceptibility to pain in spontaneously hypertensive rats (SHR). A recent study by Palma‐Rigo et al. (2010) showed that overexpression of the AT1R resulted in a decrease in BRS in both day and night. Cardiac AT1R protein and mRNA has been shown to be increased in the night with augmentation of this diurnal rhythm in SHR (Naito et al. 2002). These studies support the notion that the AT1R undergoes a cyclic variation during a 24‐h cycle in the brain as well as in other tissues. The fact that the AT1R expression peaks during the time when activity and sympathetic outflow is highest may suggest that changes is tissue renin–angiotensin system components are upstream from sympatho‐excitation in the normal state. Since the central AT1R increases in the post‐MI state and diurnal variability is lost, this would potentially contribute to a sympathetically mediated worsening of the heart failure state and to arrhythmogenesis.

While we did not measure protein changes over the first 2 weeks post‐MI, the fact that EF was not significantly different in MI mice compared to sham until the 4‐week time point may suggest a contribution of the central renin–angiotensin system to the loss in circadian variability for the hemodynamic parameters. AT1R expression has been shown to be increased in the suprachiasmatic nucleus (SCN) of Dahl salt‐sensitive rats (Wang et al. 2003). Interestingly, in this study, diurnal changes in AT1R expression were not seen in the hypothalamus, an area containing the SCN. In an interesting study by Brown et al. (2008), neurons in the SCN were depolarized by relatively high doses of Ang II. While these investigators did not measure AT1R protein or use specific antagonists, they did provide evidence for activation of GABAergic neurons in the SCN by Ang II. In an interesting and relevant study by Li et al. (2007) clock genes and the AT1R gene was measured in the SCN of normal rats and of rats with chronic sino‐aortic denervation (SAD). AT1R mRNA was markedly elevated during the light period in SAD thus reducing the 24‐h variability. While these investigators did not look outside the SCN it is of interest to speculate that the loss of circadian variability of both protein expression and hemodynamic responses is a broad phenomenon contributing to sympatho‐excitation.

While this study is suggestive of a role for Ang II signaling through the AT1R in the genesis of blood pressure circadian rhythm and its loss in animals with chronic heart failure, it is difficult to determine cause and effect in these experiments. It is certainly possible that increased sympathetic nerve activity (especially targeting the kidney) generated centrally is responsible for an upregulation in AT1R expression in the RVLM. It is equally possible that the augmented Ang II content in the CNS (Zucker et al. 2001) in chronic heart failure evokes a positive feedback to upregulate the AT1R (Liu et al. 2008; Mitra et al. 2010; Haack et al. 2012, 2013) in many areas of the CNS. While we did not see diurnal changes in AT1R expression in the hypothalamus, this could be due to the fact that the SCN is small in comparison with the entire hypothalamus; so, a dilution of the protein may have obscured a positive finding. In this regard, a limitation of this study is that larger areas of brainstem and hypothalamus were used to analyze tissue protein rather than discrete micropunches of relevant autonomic nuclei due, in part, to the difficulty of harvesting these areas from mice.

We measured gp91^phox^ protein as a surrogate for oxidative stress in the brainstem. We did not actually measure oxidative stress or antioxidant enzymes. Therefore, the link between AT1R signaling and a Nox2‐dependent increase in superoxide is circumstantial. Previous studies from this laboratory have confirmed increases in AT1R expression along with multiple NADPH oxidase subunits in the RVLM of rabbits with pacing–induced heart failure (Gao et al. 2005; Liu et al. 2008). Superoxide was also increased in these animals along with sympathetic nerve activity. Nevertheless, the role of oxidant stress in mediating changes in AT1R expression through transcriptional regulation (Thomas et al. 2004; Li et al. 2007) in both SCN and RVLM needs to be evaluated in the heart failure state.

In summary, the results of this study show that during CHF there is a disruption in diurnal variability in BP, HR, and BRS, which occurs in concert with abnormal diurnal variation in AT1R protein and message in the brainstem of CHF mice.

## Conflict of Interest

There are no conflicts of interest to declare.
